# Supplementary material for: A large invasive consumer reduces coastal ecosystem resilience by disabling positive species interactions
Source: Nat Commun. 2021 Nov 1;12:6290. doi: 10.1038/s41467-021-26504-4 (PMC8560935; doi:10.1038/s41467-021-26504-4)
Supplement: Supplementary file 1 — Supplementary Information File [file 41467_2021_26504_MOESM1_ESM.pdf]

A large invasive consumer reduces coastal ecosystem resilience by disabling positive species interactions

Supplementary Information

Supplementary Methods

Initial observations of hogs consuming ribbed mussels came from examination of feces in marshes on Sapelo Island. We conducted a haphazard hog feces count in five marshes over the course of two summers within the Sapelo Island National Estuarine Research Reserve. Every time we encountered hog feces, we examined the contents to determine if there were any ribbed mussel shell fragments and recorded a presence/absence as appropriate. Fecal samples already examined were easy to distinguish between new samples, because we broke apart feces with a stick to examine contents. Results of these surveys were simply summarized as the proportion of fecal groupings that had ribbed mussel shells (Supplementary Table 1).

To determine whether the presence of cordgrass influenced hog predation intensity on ribbed mussels, we conducted a mussel transplant experiment at Kenan Field marsh and Miller Pump marsh within the Sapelo Island NERR. We created three sets of experimental mussel beds (3 mounds per treatment per trial, 20 ribbed mussels tightly packed into a 20 x 20cm mound) in two treatments: 1) in a cordgrass patch and 2) in a mud flat within 3 m adjacent to the patch at the same distance (at least 20 m) from the upland border of the marsh. The number of mussels remaining in each mound was recorded after 1, 3, and 10 days. This experiment was repeated 6 times, 3 in 2013 and 3 in 2014 (total n=18 per treatment). The number of mussels that survived was analyzed with a generalized linear model, fit with quasibinomial error structure, with site and location in marsh (patch vs mudflat) as predictors in a factorial ANOVA framework. We

evaluated assumptions of normality of residuals using QQ plots and Shapiro-Wilks test for normality and found no violations. We also used cordgrass-patch mussel depletion data to inform hog predation behaviors in our marsh recovery model (Fig 5).

To test for the long term implications of hog trampling and mussel predation, we combine a spatial marsh recovery model roughly based on [1], with a simple non-spatial exploitation model that follows a classical setup for the exploitation of a prey species by a predator. We model how hog density *H* affects the cover of mussels *M* in a salt marsh which indirectly affects the ability of a marsh to recover form drought-induced die off. Hence, for simplicity, we presume that the cover of the vegetation is proportional to that of mussels. We further presume that mussel density in the marsh follows a logistic growth rate where *r* is the intrinsic growth rate and *K* is the carrying capacity. Predation by hogs on mussels is modelled as a linear function of both hog and mussel density: *d(M)M H*, where *d(M)* describes the rate of predation per mussel and per hog, which is a function of mussel density as hogs focus on remaining mussels once mussel density decreases, increasing predation rates in a pattern we found in our mussel transplant experiment (Supp Fig 3b). *d(M)* would include both the direct predation effects as well as mortality induced by trampling of the mussels by the hogs. The model can be described as:

$$\frac{d(M)}{dt} = r\left(1 - \frac{M}{K}\right)M - d(M)M\,H$$

Here we model how the density of mussel decreases with increasing hog population sizes, analyzing three models. The first model does not presume any focused hog predation on mussels, and *d(M)* equals to a constant *d*, i.e. it combines the effects of accidental encounters of mussels, including both predation and trampling effects. The second model presumes complete focusing of hogs on the remaining mussels, which is described here as *d(M) = d/M.* This modeled

behavior is most similar to predation patterns we observed in our mussel transplant experiment, where hog predation pressure on mussels was highest when mussel beds were nearly depleted. In this case, the total predation pressure on the mussel population reduces to dH. The third model presumes partial focusing, meaning that the predation pressure on individual mussels increases with increasing mussel density. This modeled behavior represents a combination of our field observations and information from the literature that suggests coastal hog populations primarily feed on acorns, and is less than proportional as hogs switch to their primary food sources. Here, $d(M)$ is given by $d / (M+a)$, where a is the constant lowering the impact of focusing on the mussels. We determined how long the system would require to return to 95% of full cover if hog predation would cease (i.e., recovery time), using the model described in [1]. The mussels would allow for a partial vegetation survival in the area which is linearly related to fraction of mussels remaining relative to their maximal biomass K. Vegetation is allowed to expand from the mussel pockets to fill the area, simulating marsh recovery dynamics. These models are principally theoretical, and not based on quantifications of growth, mortality, and spatial spread. Hence the model predictions should be used in a comparative way. Parameters used in the current model were $r = 1$, $K = 100$, $d = 10$, $a = 20$ (note that a is only used in model three, partial focusing).

Supplementary Note 1

First, we found strong evidence that feral hogs consume ribbed mussels readily when then enter salt marshes from the fecal survey (Supp Table 1). Over two summers, we examined 190 individual “fecal events,” defined here as a grouping of hog droppings within a meter of each other. Of the 190 fecal events examined across 5 sites, 82.1% had ribbed mussel shells present.

Further experiments supported our large-scale observations that the presence of hogs decreases the survivorship and abundance of both cordgrass and mussels. A mussel transplant experiment at two sites on Sapelo Island, GA revealed that ribbed mussels are more vulnerable to predation when transplanted into a patch of cordgrass rather than adjacent open mudflats ($n = 16$ transplants per site per condition). Ten days after transplanting, mussel survival was 25% lower in cordgrass patches (38.8 ± 0.09% survival) than in exposed mudflats (63.1 ± 0.07 % survival) at our two sites (Supplementary Fig. 3, Supplementary Table 6; ANOVA: $F_{1,28} = 4.6$, $p = 0.04$).

Additionally the total unvegetated area (i.e., mudflat cover) at each surveyed site is highest in high hog activity marshes (high hog activity = 517 ± 135.8 m^2, low hog activity = 246.1 ± 165.3 m^2; mud area: $X^2 = 1.2$, df = 2, $p = 0.332$, Supplementary Fig. 1, Supplementary Table 2c), supporting our hog activity level classification[2].

Post-hoc Tukey’s contrasts from our six-site mussel abundance survey showed strong significant differences ($p < 0.0001$) in mussel densities between all comparisons except for creekbank vs marsh platform mussel densities in high hog access marshes ($p = 0.9986$), which were not different from each other likely because hogs reduced mussel densities in all areas of the marsh (Supplementary Table 3).

Supplementary Table 1. Results from hog feces survey at 4 marshes in 2013 and 2014 on Sapelo Island, GA. Total number of feces found with ribbed mussel, *G. demissa* shells compared to the total number of fecal groupings found in total at each marsh site.

**Site**	**Feces with shells**	**Total feces found**
Cabretta	42	49
Chocolate Factory	43	51
Kenan Field	43	51
Miller Pump	24	33
Raccoon Bluff	4	6
**Total**	**156**	**190**

Supplementary Table 2. Analysis of deviance (Type II Wald F Test with Kenward-Rogers df) table for Figure 1, exclusion effects on patch recovery on two sites in Sapelo Island NERR.

Term	F	dF	dF residual	P-value
Sampling Date	171.594	1	148	< 0.001
Exclusion Treatment	96.68	1	148	< 0.001
Sampling Date * Treatment	26.353	1	148	< 0.001

Supplementary Table 3. Analysis of deviance table (Type II Wald F tests with Kenward-Roger df) for Figure 2 (a)cordgrass biomass, (b)mussel density, and (c) crab density responses to Hog x Mussel manipulation

(a) Mean final cordgrass biomass (exact Hog Exclusion p value = 0.00004981)

Term	F	dF	dF residual	P-value
Hog Exclusion Treatment	21.373	1	35	< 0.001
Mussel Treatment	2.77	1	35	0.1
Exclusion * Mussels	5.395	1	35	0.026

(b) Mean final mussel density (exact p values, in descending order = 3.747 x 10^{-9}, 2.818 x 10^{-8}, 4.519 x 10^{-8})

Term	F	dF	dF residual	P-value
Hog Exclusion Treatment	60.709	1	35	< 0.001
Mussel Treatment	50.446	1	35	< 0.001
Exclusion * Mussels	48.213	1	35	< 0.001

(c) Mean final crab burrow density (exact hog exclusion p value = 0.000004895, exact mussel treatment p value = 0.0004914)

Term	F	dF	dF residual	P-value
Hog Exclusion Treatment	29.071	1	35	< 0.001
Mussel Treatment	14.763	1	35	< 0.001
Exclusion * Mussels	6.366	1	35	0.016

Supplementary Table 4. Analysis of deviance (Type II Wald Chisq tests) tables for Figure 2 and Supplementary Figure 1

a) Total number of patches per site

Term	Chi-sq	dF	P-value
Hog activity level	15.292	2	0.0004779

b) Mean number of mussels per 120 sq m transect

Term	Chi-sq	dF	P-value
Hog activity level	429.199	1	< 0.001
Marsh Location	165.816	1	< 0.001
Hog activity level * Marsh Location	46.711	1	< 0.001

c) Mean patch area per site

Term	Chi-sq	dF	P-value
Hog activity level	10.241	2	0.006

d) Percent association between mussels and cordgrass (actual hog activity level p value = 0.0000000883)

Term	Chi-sq	dF	P-value
Hog activity level	28.603	1	< 0.001
Marsh Location	4.877	1	0.027
Hog activity level * Marsh Location	0.026	1	0.87

Supplementary Table 5. Ribbed mussel (*Geukensia demissa*) survey data from 3 marshes with and without hogs on Sapelo Island GA. Surveys were conducted both along creek heads, adjacent to marsh creeks, and in the marsh platform, at least 20m from creeks. Total number of mussel mounds and total mussels per marsh were much higher in hog-free marshes while mean area of mounds was higher in no hog marshes. Additionally, in no hog marshes mussel association with cordgrass was nearly 100% while mussels in hog-accessed marshes had much lower association rates. More mussels were on the marsh platform and in defunct (partially destroyed) mounds in marshes with hog activity. All comparisons between hog and no hog marshes, nested within location, were statistically different from each other

	**location within marsh**	**total mussel mounds**	**mussels per 120 m^2**	**% of transect covered by mussels**	**% mussels associated w cordgrass**	**% mussels on defunct mounds**	**% mussels on healthy mounds**	**total singleton mussels**
No Hog	creek head	62 ± 9.2	2079.7 ± 85.8	7.2 ± 0.5%	99.9 ± 0.02%	0 ± 0%	99.9 ± 0.07%	3
Hog	creek head	55.7 ± 2.0	111.3 ± 21.8	0.47 ± 0.01%	70.2 ± 8.83%	77.5 ± 3.78%	0 ± 0%	96
No Hog	platform	35 ± 4.6	345.7 ± 59.4	4.5 ± 0.15%	99.1 ± 0.88%	0 ± 0%	97.3 ± 0.26%	12
Hog	platform	14.3 ± 4.3	30.3 ± 8.7	0.39 ± 0.01%	49.8 ± 18.7%	78.7 ± 8.2%	0 ± 0%	27

Supplementary Table 6. Analysis of deviance (Type II test) table with Pearson residuals for Supp Fig 3 mussel transplant experiment.

	**Sum Sq**	**df**	**F value**	**P-value**
Location in marsh	1.9232	1	4.5568	0.041
Site	0.0332	1	0.0788	0.781
Location*Site	0.2270	1	0.5379	0.469
Residuals	11.8635	28		

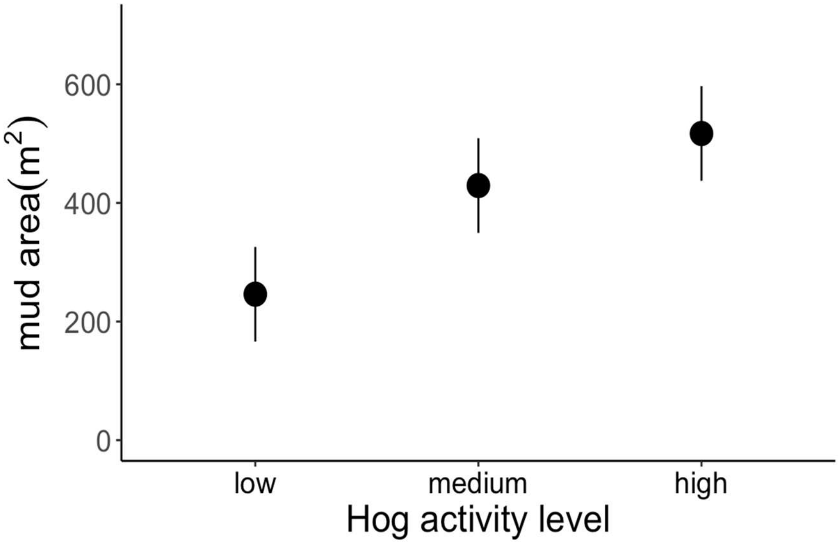

Supplementary Figure 1. Mean and standard error area in m^2 of mud (unvegetated marsh) per flight from 14 marshes in Georgia and Florida.

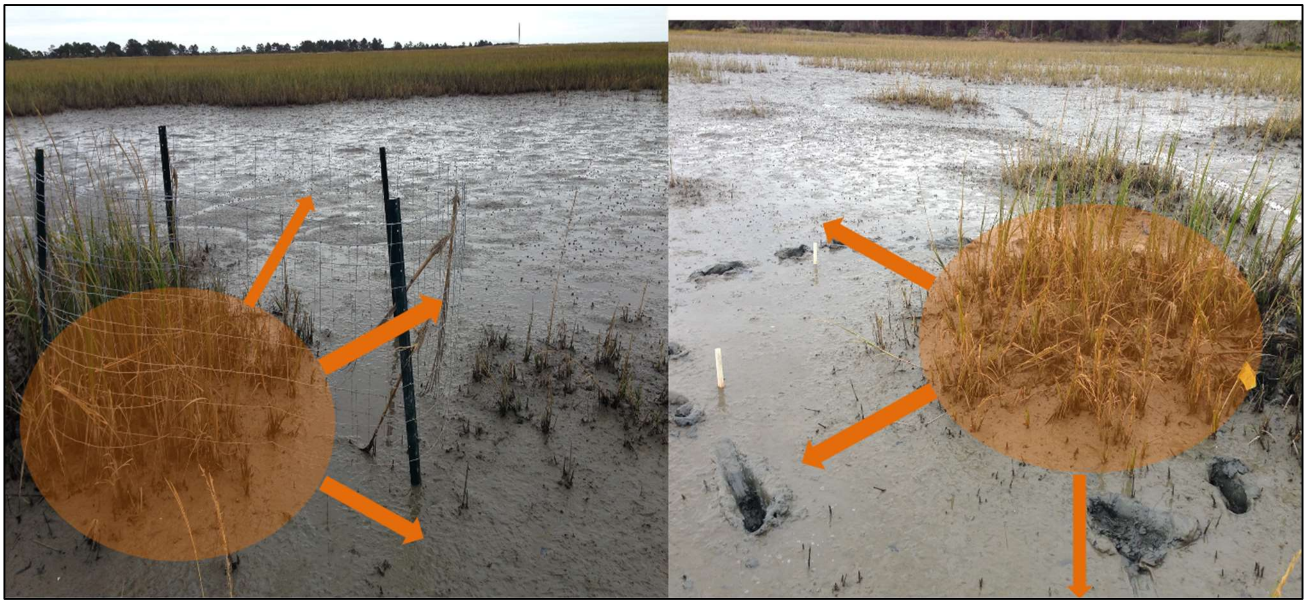

Supplementary Figure 2. Hog exclusion (left) and control (right) plots for patch recovery experiment on Sapelo Island GA. Plots were started at ~50% plant cover at the beginning of the experiment but were not manipulated pre-experiment. Percent cover was calculated at each sampling date by using a gridded quadrat over the whole plot. Orange circles and arrows represent initial patches and direction of recolonization.

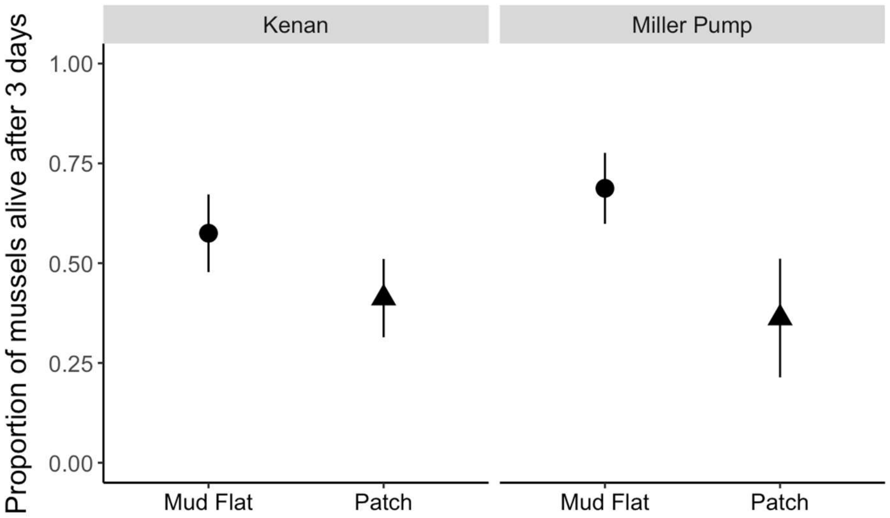

a.

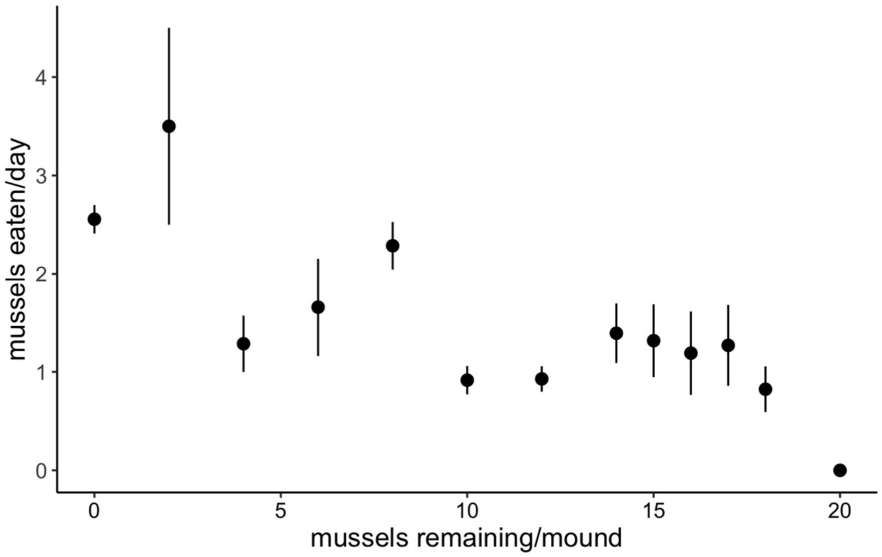

b.

Supplementary Figure 3. (a) Transplanted ribbed mussel mounds (20 mussels/mound) had higher survival rates after 3 days in bare, exposed mud flats (circle with standard error bars) than in cordgrass patches (triangle with standard error bars) when hogs were present at two sites in Sapelo Island NERR, suggesting that cordgrass presence increases the negative effect of hogs on mussel mounds. (b) Number of mussels consumed per day (mean and standard error shown as

points and error bars) was highest with the lowest mussel densities (i.e. already depleted mounds). n = 18 per treatment per site

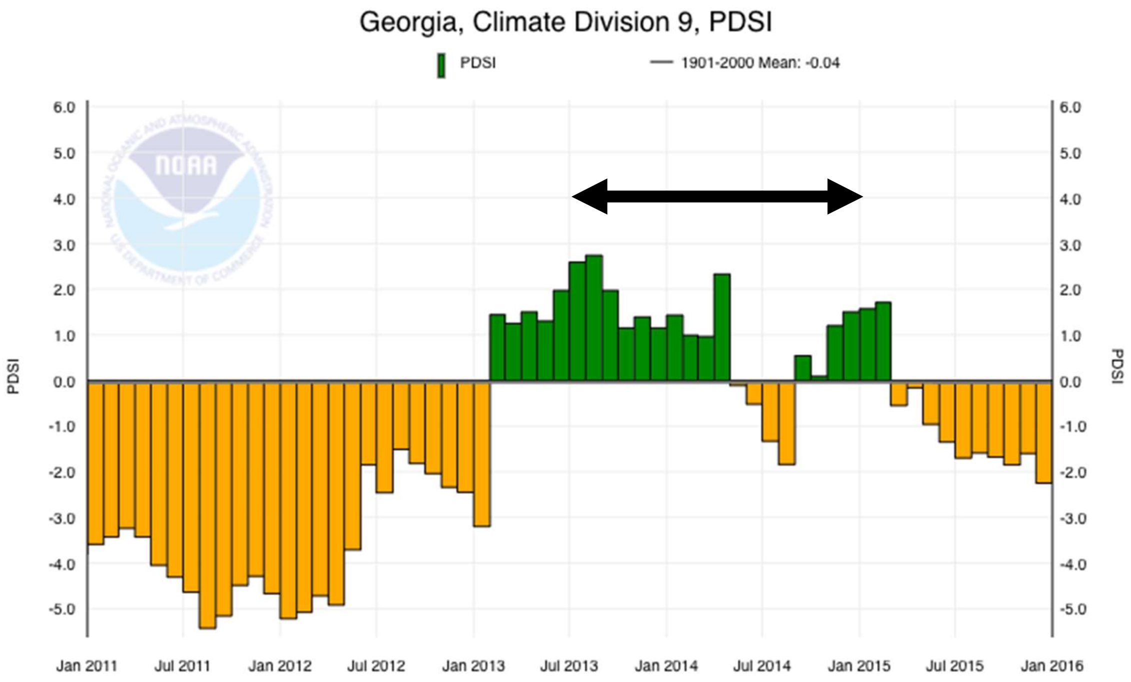

Supplementary Figure 4. Palmer Drought Severity Index (PDSI) generated from NOAA data for Georgia Climate Division 9 (SE Georgia, location of experiments) indicating that the duration of experiments, depicted with the black arrow, occurred during a drought recovery time period. PDSI was in the extreme drought range for over 2 years prior to our experiments.

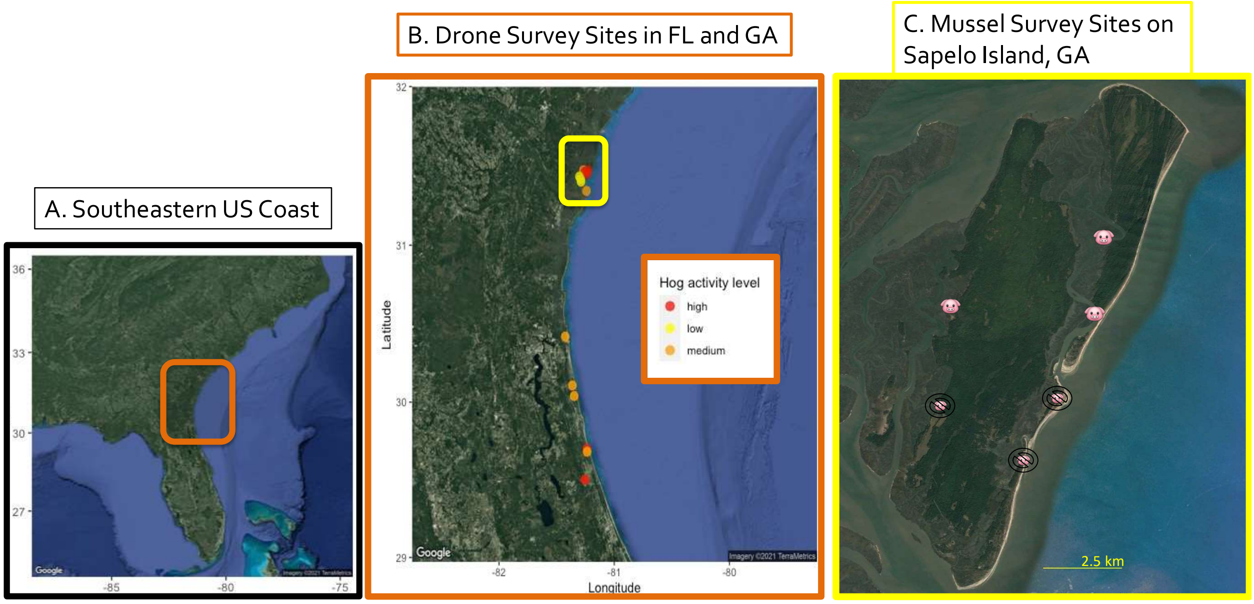

Supplementary Figure 5. (A) Sites were located in the southeastern USA, inside of the orange box (B) Drone site map showing drone survey locations, with Sapelo Island in the yellow box. (C) Sites within the Sapelo Island NERR for mussel density survey. Sites where hogs had access into (pig icon) are located further away from population centers on the southern side of Sapelo Island. Sites where no evidence of hog access was found (pig icon with crossed circle) had consistently higher mussel densities on both creekbanks and marsh platforms.

Supplementary References

1. Guichard, F., Halpin, P. M., Allison, G. W., Lubchenco, J. & Menge, B. A. Mussel Disturbance Dynamics: Signatures of Oceanographic Forcing from Local Interactions. *Am. Nat.* **161**, 889–904 (2003).
2. Sharp, S. J. & Angelini, C. The role of landscape composition and disturbance type in mediating salt marsh resilience to feral hog invasion. *Biol. Invasions* (2019) doi:10.1007/s10530-019-02018-5.
