# Supplementary material for: A large invasive consumer reduces coastal ecosystem resilience by disabling positive species interactions
Source: Nat Commun. 2021 Nov 1;12:6290. doi: 10.1038/s41467-021-26504-4 (PMC8560935; doi:10.1038/s41467-021-26504-4)
Supplement: Supplementary file 3 — Reporting Summary [file 41467_2021_26504_MOESM3_ESM.pdf]

nature research

Corresponding author(s): Marc J S Hensel

Last updated by author(s): Aug 20, 2021

Reporting Summary

Nature Research wishes to improve the reproducibility of the work that we publish. This form provides structure for consistency and transparency in reporting. For further information on Nature Research policies, see our Editorial Policies and the Editorial Policy Checklist.

Statistics

For all statistical analyses, confirm that the following items are present in the figure legend, table legend, main text, or Methods section.

n/a	Confirmed	
☐	☒	The exact sample size (*n*) for each experimental group/condition, given as a discrete number and unit of measurement
☐	☒	A statement on whether measurements were taken from distinct samples or whether the same sample was measured repeatedly
☐	☒	The statistical test(s) used AND whether they are one- or two-sided<br>*Only common tests should be described solely by name; describe more complex techniques in the Methods section.*
☐	☒	A description of all covariates tested
☐	☒	A description of any assumptions or corrections, such as tests of normality and adjustment for multiple comparisons
☐	☒	A full description of the statistical parameters including central tendency (e.g. means) or other basic estimates (e.g. regression coefficient) AND variation (e.g. standard deviation) or associated estimates of uncertainty (e.g. confidence intervals)
☐	☒	For null hypothesis testing, the test statistic (e.g. *F*, *t*, *r*) with confidence intervals, effect sizes, degrees of freedom and *P* value noted<br>*Give P values as exact values whenever suitable.*
☒	☐	For Bayesian analysis, information on the choice of priors and Markov chain Monte Carlo settings
☐	☒	For hierarchical and complex designs, identification of the appropriate level for tests and full reporting of outcomes
☒	☐	Estimates of effect sizes (e.g. Cohen's *d*, Pearson's *r*), indicating how they were calculated

Our web collection on statistics for biologists contains articles on many of the points above.

Software and code

Policy information about availability of computer code

Data collection	All data was collected in the field from experiments, or was modeled. ImageJ (v1.48) and PhotoscanPro (v1.2) were used to collect drone image data. Data and code used to conduct analyses and generate figures are available at www.github.com/mhensel/HogsInTheMarsh or DOI: 10.5281/zenodo.5225047
Data analysis	R Statistical Programming Software (R version 4.1.0) was used for all analyses. R packages used are all properly cited in the methods section: lme4 (v1.1-27.1), lmerTest (v3.1-3), emmeans(v1.6.2-1), and dHARMA (v0.4.3).

For manuscripts utilizing custom algorithms or software that are central to the research but not yet described in published literature, software must be made available to editors and reviewers. We strongly encourage code deposition in a community repository (e.g. GitHub). See the Nature Research guidelines for submitting code & software for further information.

Data

Policy information about availability of data

All manuscripts must include a data availability statement. This statement should provide the following information, where applicable:

- Accession codes, unique identifiers, or web links for publicly available datasets
- A list of figures that have associated raw data
- A description of any restrictions on data availability

Datasets that support the analyses and figures of this study have been deposited in the public repository: https://github.com/mhensel/HogsInTheMarsh or DOI: 10.5281/zenodo.5225047

Field-specific reporting

Please select the one below that is the best fit for your research. If you are not sure, read the appropriate sections before making your selection.

☐ Life sciences ☐ Behavioural & social sciences ☒ Ecological, evolutionary & environmental sciences

For a reference copy of the document with all sections, see nature.com/documents/nr-reporting-summary-flat.pdf

Ecological, evolutionary & environmental sciences study design

All studies must disclose on these points even when the disclosure is negative.

Study description	This study utilized field experiments, geographical surveys (on the ground and drone based), and mathematical models to describe how feral hogs affect the recovery and resilience of southeastern US salt marshes. Here I will briefly describe each aspect of the study:<br>To determine if there was a relationship between hog activity and salt marsh fragmentation (i.e. structure of remnant cordgrass patches) in the southeastern US, we conducted a drone survey recording the number and size of cordgrass patches in 10 marshes in Georgia and Florida that varied in hog activity. To determine if there was a large-scale correlation between hog activity and ribbed mussel densities, we conducted mussel density surveys at six marsh sites on Sapelo Island, GA. We conducted two experiments to determine how hogs affect positive interactions in salt marshes. First, to determine whether cordgrass mediates hog predation intensity on ribbed mussels, we conducted a two-week mussel transplant experiment in cordgrass patches and adjacent bare mudflat in two sites in two different years. Second, to test the hypothesis that hogs alter the strength of the positive effects of mussels on cordgrass and other associated marsh organisms, we conducted a three year two-factor experiment manipulating the presence of both mussels and hogs using exclusion cages (n=8). We also tested the causal hypothesis that hog disturbance stalls the recovery and outgrowth of salt marsh grasses into surrounding mudflats with a hog exclusion experiment of recovering patches during marsh revegetation after years of severe drought (n=10). Last, we integrated hog predation of mussels into an existing marsh recovery mathematical model to compute the time the vegetation needs to recolonize the landscape following a disturbance that removes all vegetation outside of the mussel patches.
Research sample	Response variables for this study were generally aspects of salt marsh plant or animal communities, thus were meant to represent a salt marsh cordgrass, crab, or mussel population in the coastal southeastern US. Cordgrass was the only organism removed from the marsh as a sample but the following organisms were measured: cordgrass Spartina alterniflora, fiddler crabs Uca sp, mud crabs, Eurytium limosum and Panopeus herbstii, purple marsh crabs Sesarma reticulatum, ribbed mussels Geukensia demissa. These samples were chosen to most accurately represent potential changes in the salt marsh invertebrate community due to hog activities.
Sampling strategy	The sampling strategy was to collect data to best represent field patterns while minimizing local disturbance and visits to sites. Field experiments (n = 20, 16), drone surveys (n = 14), and mussel surveys (n = 6) sampling sizes were limited by cost and time allocation. Field experiments ran for at least 2 years, while surveys were conducted once
Data collection	All data was collected in the field using visual techniques or, in the case of the drone survey, collected on video camera and analyzed on a computer later on. All field data was collected visually by one of the following researchers: M. Hensel, E. Hensel, S. Sharp, S. Crotty via pencil and notebook while in the field.<br><br>We conducted a drone survey recording the number and size of cordgrass patches in 14 marshes in Georgia and Florida that varied in hog activity. Sites were chosen within the Southeastern US to minimize variability across large scales, then standardized for size (between 0.6 and 1 km2), and variation in estimated hog activity. Using a DJI Phantom 3 drone with a GoPro Session camera attached, we surveyed three 15000m2 areas (approximately 50 m wide, 300 m long), at least 500m apart, in each salt marsh sites (30 total flights). Drone path length, which determines area covered, as well as the height of the drone was standardized (30m high) to control the total area covered for each drone flight. We created a photo mosaic of each flight using PhotoscanPro. We calculated total number of live cordgrass patches and mean patch size for each of the flights, scaling each mosaic with a known, marked area on the ground, and manually circling and measuring patches using ImageJ. Each image had 10m long markings as a scale bar<br><br>In our patch recovery experiment, we marked 16 replicated 2x2m plots on the edge of similarly sized patches (between 9m2 and 16m2) within a bare mud-remnant patch matrix, where no two patches were closer than 5 m from each other in May 2013 at Kenan Field and Miller Pump (~4 km away) marshes in the Sapelo Island NERR. We set up each plot to have the same initial starting percent cover (50%), in order to properly capture recolonization and recovery rate. Mesh size of the hog wire was 20 cm2 and cages were 1.5m tall, allowing access to all marsh species including other large mobile predators like blue crabs, fish, and raccoons. We found evidence (e.g. raccoon tracks, crushed mussel shells) that these other predators were allowed equal access to caged and uncaged plots. The only other exclusions were likely deer, horses or cows, which are not known to consume marsh organisms and only occur at very low densities in our study sites 90. In this study, we observed no buildup of wrack material and hog exclusions with smaller mesh (2cm2) have shown no evidence of flow artifacts. For each plot we standardized initial plant density and percent cover (130-150 cordgrass stems/m2; 50% cover) and selected plots with nearly identical initial invertebrate densities (5-10 fiddler crab Uca sp. burrows/m2, 0-25 snails Littoraria irrorata/m2, and 0-10 ribbed mussel Geukensia demissa /m2) to ensure there were no differences between plots and within sites. Initial plant cover was not manipulated at the beginning of the experiment, rather, we specifically selected the same sized patches with similar stem densities and explicitly marked plots so that each plot began at 50% cover, with half of the plot covering the vegetated patch and the other half covering unvegetated mudflat, i.e., where cordgrass recovery would occur. Over the course of the experiment, we recorded percent cordgrass cover using a gridded quadrat. Final data on this experiment was recorded in July 2015 after 24 months in the field.<br><br>We conducted mussel density surveys at six marsh sites on Sapelo Island, GA in 2018. Within each marsh, we selected 120m2

transects in each of two area types: the creek head, where water first enters the marsh platform and mussel cover is highest 91, and the high marsh platform, zones of short-form cordgrass located 30m from the terrestrial border and >50m from the nearest creek head. Mussel surveys were conducted at three known hog-accessed and three hog-free sites. Sites were selected based on proximity to human development, a proxy for hog-access on Sapelo Island (Supp Fig 5, Georgia Department of Natural Resources pers. comm, M. Hensel pers. obvs) and were similar in elevation, creek size and number, and total marsh area. Within each 120m2 transect, we counted all singleton mussels and mussel aggregations, and measured the dimensions of each mussel and mound encountered (LxWxH). For each singleton mussel and mound, we identified whether the mussel or aggregation of mussels was associated with cordgrass through attachment by byssal threads, and categorized the local area type as marsh platform, active mussel mound, or defunct mussel mound—locations where mussel mound remnants are visible (i.e. shell fragments, bump in primary productivity, mussel pseudofeces buildup), but no mussels (or very few) remained.

We conducted a three year two-factor experiment manipulating the presence of both mussels and hogs using exclusion cages. At both sites, we selected 20, 2x2m plots in cordgrass patches and randomly assigned each to one of the following treatments: 1) Hog exclusion, no mussels, 2) Hog exclusion, mussels added, 3) Hog control, no mussels, 4) Hog control, mussels added. Mussel addition treatments consisted of four separate mussel mounds with 20 individual adult mussels per mound, reflecting natural mound densities for coastal Georgia marshes 40. Mussel addition treatments were reapplied at the end of year 1 and year 2 of the experiment as some (~2-6 mussels/cage/year) mussels inside of hog exclusion cages were consumed by raccoons or experienced mortality from transplant stress and there were no mussels left in any uncaged plots. Throughout the course of the experiment we measured live and dead grass mass, as well as marsh community structure that has been shown to be positively affected by this mutualism (i.e. fiddler crab and mud crab burrow density).

To test for the long term implications of hog trampling and mussel predation, we integrated hog predation of mussels within an existing numerical model of salt marsh recovery. We extended this model to include a mussel population, whose cover can vary between zero and a maximum standing crop (defined as one) which is equivalent to the maximal mussel cover observed in the area of about 10%. We presumed a set hog population, which exerts a certain predation pressure on the mussels, determining mussel cover. Modeled hog population predation pressure varied based on three different predation methods: no focus of hogs on mussels (i.e., haphazard consumption when hogs enter the marsh), incomplete focus of hogs on mussels, and complete hog focusing on mussels. Assuming a random distribution of mussel patches in the landscape, we then computed the time the vegetation needs to recolonize the landscape following a disturbance that removes all vegetation outside of the mussel patches, mimicking a drought-induced die-off event

Timing and spatial scale	Hog feces survey: May 2013-August 2015, data collected haphazardly, monthly across whole marshes (0.1 - 1 km2 marsh). Patch recovery exclusion experiment and mussel addition experiment: May 2013-September 2016 (August 2015 for patch recovery experiment, see exclusions below), data collected monthly during growing season, plot level data collected. Drone survey, November 2017, Mussel survey, September 2018, data collected once over two weeks, whole marsh scale data collection (0.1-1km2 area). Data collection frequency was determined by both time availability and to try to minimize disturbance to areas and minimize spreading the scent of humans around areas where hogs visit.
Data exclusions	6 months before the planned end of the patch recovery experiment (Summer 2015), University of Georgia archaeologists began work near one of our marsh sites. As human activity tends to negatively affect feral hog usage of areas, we decided to end the exclusion experiment early.
Reproducibility	Besides multiple replications of certain aspects of this study over time (e.g., hog feces survey done during two different years, mussel transplant experiment done during two different years) that we eventually pooled into major response variables, we did not attempt to reproduce experimental results. The large-scale and long-term nature of our study make reproducibility nearly impossible, as experimental treatments were applied for multiple years. We expect that our methods are complete enough for reproducibility in both the field and in analyses.
Randomization	For experimental randomization, we first laid out plots and then used random number generators to determine which treatment each plot received. For surveys (i.e., mussel survey, drone survey) we used a map to randomly select areas of marsh to sample. Each marsh itself was selected non-randomly for these surveys in order to minimize variation in marsh elevation, creek number and size, and overall marsh size. In our drone survey, geographic randomization was accounted for by selecting an equal number of high-hog and low-hog sites in both Georgia and Florida. We expect that randomization was maximized for the drone survey, but for the mussel survey we selected sites on Sapelo Island that were known (by local managers and researchers) to either have hogs or no hogs. No hog marshes were concentrated on the southern end of the island, closer to human development, while hog-accessed marshes were further north. By controlling for marsh size and utilizing our extensive knowledge of these field sites, we minimized variation in these surveyed marshes as best as we could.
Blinding	Blinding was not possible due to the low number of researchers collecting data (generally one or two) but blinding should not affect the validity of this field collected data

Did the study involve field work? ☒ Yes ☐ No

Field work, collection and transport

Field conditions	Study took place over several years, with most field work concentrated during early summer (hot, humid coastal Southeastern US conditions) and early fall (mild conditions). Experiments and surveys were conducted during a drought-recovery period for the Coastal Southeast (see Supp Materials)
Location	Experiments, mussel, and feces surveys were conducted on Sapelo Island Georgia in the Sapelo Island NERR and LTER sites (~31.4764° N, 81.2409° W). Drone surveys were conducted along the GA and FL coast in state parks or LTER sites, including at the

	Guana Tolomato-Matanzas LTER (30.0226° N, 81.3262° W), and Timucuan Ecological and Historical Preserve (30°27'14.7"N 81° 27'01.0"W). Mean tidal height was similar at all selected sites, but elevation data was not collected. Using maps we visually identified marshes with similar creek numbers and marsh sizes for all data collection.
Access & import/export	Habitat access was done in a low disturbance manner with only footprints left behind. We entered most marsh habitats from the upland forest border. The only samples that were removed from the marsh for further analyses were grass biomass samples, which were processed at the University of Georgia Marine Institute on Sapelo Island. While we did not make any contact with our study species, the feral hog, we did obtain IACUC permits for our exclusion experiments. At the genesis of this project, lead author Hensel and second author Silliman were at the University of Florida, and received UF IACUC approval (201207684) on 11/5/2013. No contact with vertebrate animals occured during the course of this experiment and we observed no entanglement of any animals in our cages.
Disturbance	Trampling was mostly contained to muddy areas (i.e., no vegetation). Data collection occured once every few months, as to minimize scientist derived disturbance in each area. Caging materials were collected one year after the experiment

Reporting for specific materials, systems and methods

We require information from authors about some types of materials, experimental systems and methods used in many studies. Here, indicate whether each material, system or method listed is relevant to your study. If you are not sure if a list item applies to your research, read the appropriate section before selecting a response.

Materials & experimental systems

n/a	Involved in the study	
☒	☐	Antibodies
☒	☐	Eukaryotic cell lines
☒	☐	Palaeontology and archaeology
☐	☒	Animals and other organisms
☒	☐	Human research participants
☒	☐	Clinical data
☒	☐	Dual use research of concern

Methods

n/a	Involved in the study	
☒	☐	ChIP-seq
☒	☐	Flow cytometry
☒	☐	MRI-based neuroimaging

Animals and other organisms

Policy information about studies involving animals; ARRIVE guidelines recommended for reporting animal research

Laboratory animals	No laboratory animals were used in this study
Wild animals	We observed the effects of feral hogs on marsh areas through exclusion experiments and drone surveys in areas with and without hogs. Besides two haphazard encounters with hogs in the field where we saw hogs and they promptly ran away, we did not make any contact with said animals during the experiment. The rest of the animal data collection was counting densities in the field of lower invertebrates like crabs, mussels, and snails.
Field-collected samples	no animal samples were collected in the field
Ethics oversight	While we did not make any contact with our study species, the feral hog Sus scrofa, we did obtain IACUC permits for our exclusion experiments. At the genesis of this project, lead author Hensel and second author Silliman were at the University of Florida, and received UF IACUC approval (201207684) on 11/5/2013. No contact with vertebrate animals occured during the course of this experiment and we observed no entanglement of any animals in our cages.

Note that full information on the approval of the study protocol must also be provided in the manuscript.
